# Breast Imaging Findings in Women with Lipedema: A Retrospective Cross-Sectional Descriptive Study

**DOI:** 10.3390/jcm14248940

**Published:** 2025-12-18

**Authors:** Elettra Fiengo, Andrea Sbarbati

**Affiliations:** 1 Studio Iris, Pomezia, 00071 Rome, Italy; 2 Department of Neurological, Biomedical and Movement Sciences, Anatomy and Histology Section, University of Verona, 37100 Verona, Italy

**Keywords:** breast imaging, fibroglandular density, microcalcifications, stromal dysfunction, extracellular matrix, connective tissue disorders

## Abstract

**Background/Objectives**: Lipedema is a chronic, progressive adipo-fascial disorder characterized by connective tissue dysfunction, fibrosis, microangiopathy, and adipose tissue proliferation. Although lipedema has traditionally been described as a regionally confined disorder, emerging evidence suggests that it may reflect a broader stromal and connective tissue dysfunction. It is therefore plausible that anatomical regions not historically associated with lipedema may also exhibit alterations consistent with this dysfunctional stromal pattern. From this perspective, breast tissue—rich in fibro-glandular and stromal components—represents a compelling model in which to investigate whether such features are present. The breast, with its complex fibro-glandular and stromal architecture, represents a physiologically plausible site of involvement; however, its structural features in lipedema have never been systematically examined. The primary aim of this study was therefore to determine whether breast tissue—rich in fibro-glandular and stromal components—shows recurrent imaging or histopathological features suggestive of lipedema-related involvement. A secondary aim was to compare the frequency of these findings with patterns typically reported in healthy screening populations. **Methods**: This retrospective cross-sectional study analyzed 62 women (mean age: 44 ± 8 years), obtained between September and November 2025, with a clinical diagnosis of lipedema who voluntarily provided breast imaging reports (ultrasound, mammography, or magnetic resonance imaging, MRI). **Results**: The findings revealed a remarkably high prevalence of fibro-glandular parenchyma (45%), multiple diffuse cysts (42%), microcalcifications (21%), and fibroadenomas (43.5%), with frequencies substantially exceeding those documented in healthy screening populations. Additional features included significant breast asymmetry or tuberous morphology (6%), reactive or sclero-lipomatous lymph nodes (19%), and recurrent stromal hyperplasia on biopsy. Histological evaluations (n = 9) consistently showed fibroproliferative alterations, including stromal hypercellularity, adenosis, fibroepithelial lesions, apocrine metaplasia, and pseudoangiomatous stromal hyperplasia, suggesting a shared extracellular matrix-related dysplastic phenotype between lipedema-affected breast tissue and peripheral adipose tissue. **Conclusions**: These findings support the hypothesis that lipedema may express a characteristic breast phenotype driven by stromal and extracellular matrix dysregulation. If confirmed in larger controlled studies, these recurrent alterations could contribute to improved diagnostic frameworks and raise awareness of lipedema as a systemic connective tissue disorder with underrecognized breast manifestations.

## 1. Introduction

Lipedema has traditionally been described as an abnormal, disproportionate, and symmetrical accumulation of adipose tissue, predominantly affecting the lower limbs and, to a lesser extent, the upper limbs, and typically accompanied by pain, inflammation, and fibrosis [1]. Etiopathogenetic hypotheses have focused on genetic [2], hormonal [3], vascular [4], and inflammatory factors [5]. This diagnostic description—historically developed to differentiate lipedema from obesity, lymphedema, and other vascular disorders—has played a central role in shaping the disease’s nosology. However, there is currently no solid evidence demonstrating that lipedema is truly confined to a single anatomical district.

An increasing body of research suggests that lipedema is better understood as an adipo-fascial, stromal, and connective tissue disorder characterized by extracellular matrix (ECM) abnormalities, interstitial fluid dysfunction, microangiopathy, and aberrant fibro-adipose proliferation [4,6]. Recent findings support the hypothesis that adipose disorders such as lipedema and Dercum’s disease involve fascial remodeling that extends beyond adipocyte hypertrophy [7]. Prior studies in individuals with co-occurring adipose disorders and hypermobility spectrum conditions have demonstrated an association between immune dysregulation and increased thickness of the pretibial superficial fascia [7]. This suggests that immune dysregulation contributes to superficial fascial changes in hypermobile Ehlers–Danlos syndrome (hEDS), hypermobility spectrum disorders (HSD), and in adipose tissue disorders occurring concomitantly. Both lipedema and Dercum’s disease are associated with chronic, low-grade inflammation mediated by M2 macrophages. These cells promote angiogenesis, interstitial fluid accumulation, and ECM remodeling. The presence of M2-mediated inflammation supports the concept that these conditions are not isolated adipose disorders but, rather, systemic fascial pathologies [8]. Previous work by our group has shown that lipedema shares several clinical and connective-tissue–related features with hypermobility spectrum disorders (HSD), supporting the hypothesis of a systemic extracellular matrix dysfunction [9]. It is therefore plausible to hypothesize that anatomical regions not traditionally associated with lipedema may also exhibit alterations consistent with this dysfunctional stromal pattern. From this perspective, breast tissue—rich in fibroglandular and stromal components—represents a compelling model to investigate whether such features are present.

In clinical practice, however, breast alterations in women with lipedema have not yet been systematically studied. This knowledge gap is compounded by the fact that, according to patient reports and clinical review, radiologists and breast specialists are often unfamiliar with lipedema, leading to uncertainty and a lack of tailored diagnostic guidance. Moreover, review of the collected imaging reports revealed that only rarely did the diagnostic workup include a structured medical history capable of detecting relevant coexisting conditions—such as endometriosis, fibromyalgia, joint hypermobility, psoriasis, connective tissue diseases, Raynaud phenomenon, celiac disease, non-Hodgkin lymphoma, glomerulonephritis, sebaceous cysts, uterine fibroids, diffuse joint pain, atrial septal aneurysm, recurrent umbilical hernia, prior tonsillectomy, adenectomy, cholecystectomy, prurigo nodularis, varicocele, or gastroesophageal reflux disease—which further reinforces the hypothesis of systemic connective tissue involvement.

It is reasonable to assume that, had clinical history been consistently and systematically collected, the existing dataset on the multisystem manifestations of lipedema would already be far more comprehensive. The absence of such structured assessment contributes to the fragmented understanding of the disease and slows progress in elucidating its full pathophysiology.

In light of these considerations, the present study aims to systematically describe breast alterations observed in a cohort of women with a diagnosis of lipedema, with the goal of examining whether such findings align with the stromal and connective tissue dysfunction model that characterizes the condition. To our knowledge, this represents the first observational analysis specifically dedicated to exploring the breast phenotype in lipedema.

## 2. Materials and Methods

We conducted a retrospective, cross-sectional, descriptive observational study including 62 patients (mean age 44 ± 8 years) with a clinical diagnosis of lipedema who provided documentation of previous breast imaging examinations (mammography and/or ultrasound). Participants were recruited through social media platforms. At the time of data collection, participants provided identifying information, which was stored separately; for the purposes of the study, all data were anonymized by assigning each participant a numerical code. All participants received an information sheet outlining the aims and observational nature of the study and were informed that participation was voluntary. Women who agreed to participate voluntarily submitted their clinical information via e-mail and provided written informed consent for the use of their data for scientific research purposes. All imaging findings included in this study were obtained during routine clinical assessments performed between September and November 2025.

Participants who consented were asked to provide documentation related to:

- their lipedema diagnosis (clinical or diagnostic report),
- breast imaging studies (breast ultrasound, mammography, and/or magnetic resonance imaging).

### 2.1. Inclusion Criteria

Patients were included if they:

- had a clinical diagnosis of lipedema established by a specialist;
- had undergone at least one breast imaging examination (ultrasound, mammography, or MRI);
- provided copies of radiological reports and, when available, histological findings.

### 2.2. Breast Imaging Variables Collected

Breast imaging reports were reviewed, and the following findings were recorded:

- breast cysts;
- microcalcifications;
- fibroadenomas or other benign nodular lesions;
- predominance of fibroglandular tissue;
- predominance of adipose, stromal, or mixed composition;
- marked breast asymmetry or tuberous breast morphology;
- history of breast surgery (including implants and complications such as capsular contracture);
- suspicious or malignant lesions confirmed by biopsy;
- presence of reactive lymphadenopathy.

### 2.3. Statistical Analysis

Statistical analyses were performed using Microsoft Excel (Microsoft Corp., Redmond, WA, USA). Continuous variables (such as age and body mass index) are presented as mean and standard deviation (SD). Categorical variables are reported as absolute frequencies and percentages. No dedicated statistical software package was used. Body Mass Index (BMI) data were available for 31 patients; in this subgroup, the mean BMI was 25.8 ± 4. Regarding disease staging, the sample included 13 patients with stage 1 lipedema, 6 with stage 1/2, 26 with stage 2, 1 with stage 2/3, 3 with stage 3, and 1 patient with lipolymphedema. The analyses performed in this study were purely descriptive; no inferential statistical tests were conducted, and therefore, no *p*-values are reported

### 2.4. Rationale for Study Design

This study did not include a control group because its purpose was not to quantify risk differences or estimate the prevalence of breast alterations relative to a healthy population, but rather to systematically describe the findings observed within a cohort of women with lipedema. This approach is typical of cross-sectional descriptive observational studies aimed at phenotypic characterization of conditions that remain poorly understood. Given the exploratory nature of the field, the scientific priority is to document the pattern of findings rather than establish formal comparisons. Additionally, previously published international data already provide reference values for the general population, which we have reported in full. The aim is, therefore, hypothesis-generating rather than inferential.

## 3. Results

Breast symptoms were reported by a subset of participants: 14.51% experienced persistent breast pain or tension, 9.6% reported occasional discomfort, 6.4% reported symptoms associated with the menstrual cycle, and 6.4% described pain upon palpation or during imaging examinations.

**Figure 1:**
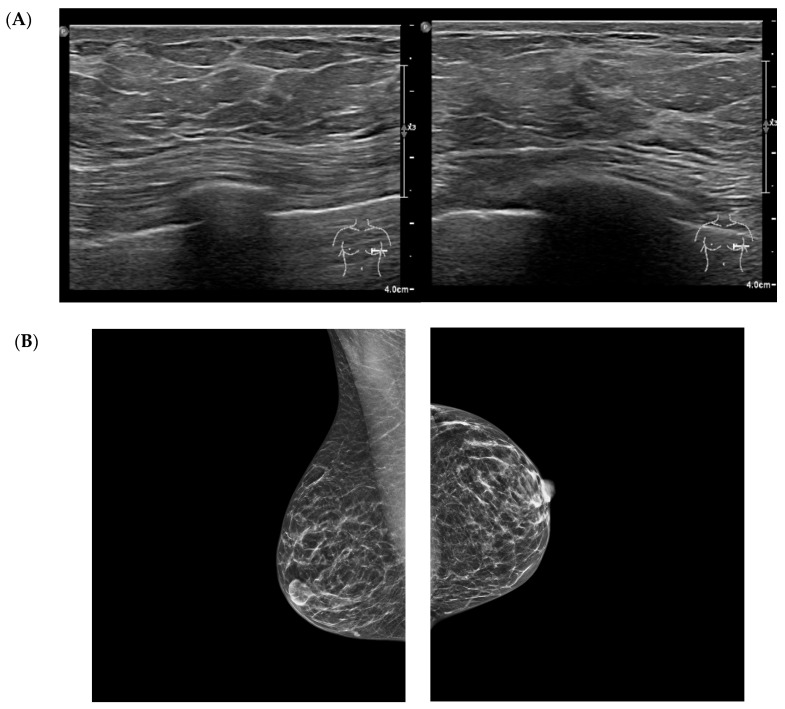
Multimodal breast imaging from a patient included in the study cohort. (**A**) high-frequency breast ultrasound (qualitative illustrative image) shows increased echogenicity of the fibrous septa, finely lobulated adipose architecture, and diffuse stromal thickening without focal lesion. (**B**) Standard mediolateral oblique and craniocaudal mammography views demonstrate diffuse fibroadipose density, and a homogeneous, non-nodular parenchymal pattern. Images were obtained during routine clinical care and anonymized for research purposes.

Regarding breast tissue composition, 45% of patients exhibited a predominantly fibro-glandular pattern characterized by a dense fibrous component. Approximately 16% demonstrated a primarily fibro-adipose pattern, while 24% showed a predominantly glandular structure. Representative multimodal breast imaging findings these tissue patterns are shown in Figure 1. Multiple and diffuse breast cysts were identified in 42% of the cohort. Calcifications or microcalcifications—mostly benign and in some cases with dystrophic appearance—were present in 21% of patients. Marked breast asymmetry or tuberous breast morphology was reported in 6%.

**Figure 2:**
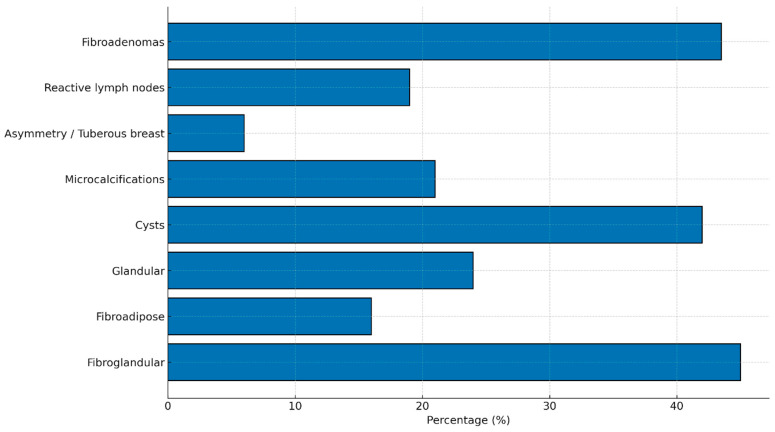
Main structural and radiological breast findings in women with lipedema. The most frequent alterations include a predominantly fibroglandular breast pattern (45%), multiple cysts (42%), fibroadenomas (43.5%), and microcalcifications (21%). Reactive lymph nodes were observed in 19% of patients, while significant asymmetry or tuberous breast morphology occurred in 6%. Data represent the distribution of key imaging findings within the study cohort.

Three patients had undergone prior breast augmentation with implants; all three developed clinically significant capsular contracture requiring medical and/or surgical management. Reactive lymph nodes, lymphadenopathy, or sclerolipomatous lymph nodes were reported in 19% of patients. Fibroadenomas were identified in 43.5% of the cohort. These findings are summarized in Figure 2.

### 3.1. Histopathological Findings

Nine biopsies were performed, revealing the following diagnoses:

Fibroepithelial lesion with focal stromal hypercellularity.

Proliferation of tubular structures lined by epithelial and myoepithelial cells, surrounded by scant stroma with lymphoplasmacytic elements (tubular adenoma).

Hyperplastic ductal epithelial fragments with rare microcalcifications and scattered stromal remnants.

Micropapillary infiltrating ductal carcinoma with foci of adenosis.

Columnar cell hyperplasia with cystic apocrine metaplasia and isolated endoluminal microcalcifications.

Breast parenchymal fragments with features of florid and sclerosing adenosis, duct ectasia, and intraductal papilloma.

Benign fibroepithelial lesion with fibroadenomatoid hyperplasia and marked pseudoangiomatous stromal hyperplasia (PASH).

### 3.2. Invasive Ductal Carcinoma

Breast parenchymal fragments with fibrocystic mastopathy, apocrine metaplasia, and stromal microcalcifications

## 4. Discussion

**Figure 3:**
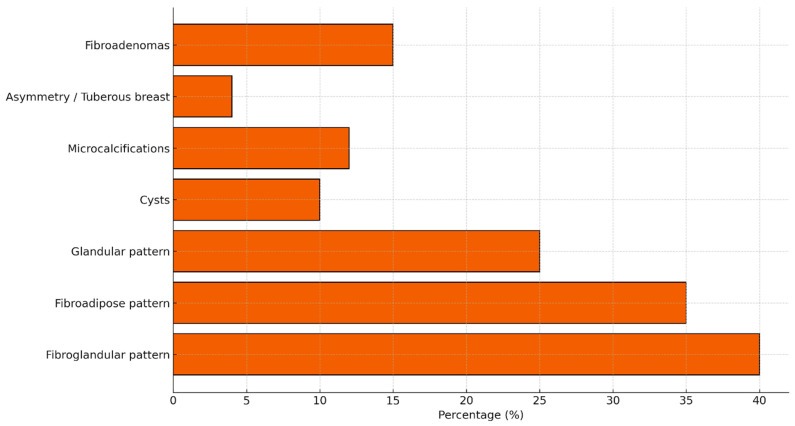
Prevalence of main structural and radiological breast findings in the general healthy population, based on published epidemiological and radiological data. Typical prevalence ranges include: fibroglandular pattern ~40%, fibroadipose ~35%, glandular ~25%, breast cysts ~10%, microcalcifications 7–12%, asymmetry ~4%, and fibroadenomas 10–20%.

The distribution of mammographic density in our cohort does not substantially differ from what is reported in the general population, in which Breast Imaging Reporting and Data System (BI-RADS) B is described in approximately 20–30% of women [10]. The predominance of a fibro-glandular pattern in the women with lipedema examined in this study is noteworthy, as this feature has not been previously characterized in the literature. As shown in Figure 3, the prevalence of a fibro-adipose pattern (BI-RADS A) in the general population ranges between 10% and 20%, depending on the cohort, and the values observed in our sample fall within this interval and do not show meaningful deviations from published data. However, breast density remains an important aspect to consider. Because breast cancers also appear radio-dense on mammography, high breast density can hinder tumor detection due to a masking effect, thereby limiting both the sensitivity and specificity of mammography [11,12,13]. Moreover, breast density is recognized as an independent risk factor for the development of breast cancer, with women who have dense breasts demonstrating up to a six-fold increased risk compared with those with fatty breasts [14,15,16]. High breast density is also associated with increased rates of false-negative examinations and interval cancers [17,18,19]. The fibro-glandular pattern observed in our lipedema cohort is consistent with the systemic phenotype of the condition, in which fibrotic nodules are frequently described in other anatomical regions. The recurrent coexistence of cysts and nodules suggests a shared mechanism involving ECM dysregulation and stromal remodeling—hallmarks of connective tissue disorders associated with lipedema. Lipedema is characterized by widespread alterations of the extracellular matrix, disorganized septal fibrosis, and chronic microinflammation. These features, which promote nodule formation in the adipose tissue of the limbs, may reasonably contribute to the recurrent presence of benign microcysts within breast parenchyma. The high prevalence of breast cysts in our cohort may reflect the same stromal instability observed in the subcutaneous tissues of individuals with lipedema, defined by fibrotic nodularity, microangiopathy, and abnormal ECM proliferation. The synchronous presence of cysts and nodules across different anatomical districts supports the hypothesis of a systemic connective tissue dysfunction rather than a localized breast disorder.

In the healthy population, studies in asymptomatic women indicate that approximately 1.5% present microcysts on ultrasound [20], whereas benign mammographic calcifications have been reported in about 9.6% of screening examinations [21,22]. Palpable breast cysts over the lifespan have been documented in approximately 7% of women in Western countries [22].

Work by Rachelle Crescenzi (2018, 2020) [23,24] demonstrated that women with lipedema exhibit significantly increased sodium content in the skin and subcutaneous adipose tissue, quantified using sodium magnetic resonance imaging (^23^Na MRI). Alteration of the stromal microenvironment—characterized by interstitial sodium retention, increased osmolarity, and extracellular matrix dysfunction—may theoretically promote a pro-fibrotic and pro-inflammatory milieu. These mechanisms are compatible with a higher predisposition to microcalcification formation, as observed in our cohort. Although a sodium–calcification link has not yet been demonstrated in the breast tissue of individuals with lipedema, current knowledge of connective tissue biology provides a coherent physiopathological basis.

The presence of marked asymmetries or tuberous breast morphology may reflect a congenital variant in breast morphogenesis. Several findings observed in our patients (volumetric asymmetry, differences in parenchymal development, and potential segmental hypoplasia) are compatible—although not diagnostic—with mild forms within the tuberous breast spectrum, commonly considered expressions of congenital connective tissue dysplasia. Tuberous breast is a malformation characterized by constriction of the breast base, segmental glandular hypoplasia, and thickening of periareolar fibrous septa. The asymmetries observed in some participants may therefore represent subclinical dysplastic variants; however, this study does not allow for formal diagnostic classification. This hypothesis aligns with existing literature linking connective tissue dysplasia with abnormal breast development.

Regarding patients who had undergone breast augmentation, although the number of cases was small, the finding that all three developed clinically significant capsular contracture is noteworthy. Capsular contracture (CC) is the most common complication after breast augmentation, with a reported prevalence of 5–19% in cosmetic augmentation and 19–25% in breast reconstruction [25]. CC represents a localized problem caused by an excessive fibrotic foreign-body reaction to the implant [26]. Although the precise mechanism remains unclear, it is hypothesized to involve a chronic inflammatory response driven by innate immune cells, leading to excessive collagen synthesis, fibrosis, pain, and abnormal firmness.

Within the context of lipedema and associated connective tissue disorders, this observation may reflect an underlying stromal vulnerability, characterized by dysfunctional ECM remodeling and a predisposition to abnormal capsular fibrosis. Lipedema displays a prominent extracellular matrix phenotype marked by septal fibrosis, disorganized collagen fibers, increased myofibroblast activity, and microinflammation. Because capsular contracture is similarly mediated by TGF-β signaling, myofibroblasts, and stiffened ECM, it is biologically plausible that individuals with lipedema may be more susceptible to pathological periprosthetic capsule formation. This association, however, has not been systematically studied and warrants further investigation.

The prevalence of fibroadenomas in the general population varies widely depending on cohort characteristics and diagnostic modalities. The most recent large-scale ultrasound study (n = 11,898 healthy women aged 18–40 years) reported a prevalence of 27.6% [27]. The presence of benign or reactive axillary lymph nodes is a common finding in the general population [28].

Overall, the findings from our cohort suggest that women with lipedema exhibit a surprisingly frequent constellation of breast structural alterations, consistent with a characteristic connective tissue and stromal phenotype.

Key Findings

1. Unusually high prevalence of fibro-glandular parenchyma

Nearly half of the patients (45%) exhibited a predominantly fibro-glandular breast pattern with a dense and fibrous stromal component. This finding is noteworthy because

- no studies to date have documented a similar prevalence in the general population;
- in the literature, such markedly stromal patterns are more typically associated with dysplastic conditions or specific hormone-responsive states.

In our cohort, however, the stromal/fibrous component appears as a recurrent feature, to the extent that it may represent a characteristic breast pattern in women with lipedema.

2. High frequency of cysts and microcalcifications

The prevalence of diffuse cysts (42%) and microcalcifications (21%) exceeds that reported in large screening studies of the general population.

The critical observation is not the isolated percentage but the cluster of alterations:

- densely fibro-glandular architecture,
- stromal proliferation,
- multiple cysts,
- microcalcifications.

Taken together, this constellation is consistent with a breast tissue phenotype that is hyperreactive, proliferative, and mechanically dysfunctional—conceptually analogous to the phenotype observed in lipedema-affected adipose tissue (adipocyte proliferation, fibrosis, ECM remodeling).

3. Elevated frequency of fibroadenomas

A total of 43.5% of patients presented with one or more fibroadenomas.

In the general population, the estimated prevalence ranges from 10% to 20%.

This substantial difference suggests:

- a stromal–epithelial proliferative tendency,
- instability of the ECM microenvironment,
- possible shared pathophysiological pathways with other fibro-dysplastic manifestations of connective tissue.

4. Lymphadenopathy and reactive lymph nodes

Nineteen percent of patients exhibited reactive or sclero-lipomatous lymph nodes.

When considered within the context of the microvascular and lymphatic dysfunction already described in lipedema, this finding may reflect a chronic stromal and immune response as part of the systemic disease phenotype.

5. Adverse reactions to breast implants

All three patients with breast implants developed clinically significant capsular contracture.

Given that capsular contracture is linked to:

- local immune dysregulation,
- altered ECM turnover,
- a predisposition to fibrosis,

This observation strengthens the hypothesis of an underlying connective tissue vulnerability in women with lipedema.

6. Histological findings consistent with diffuse stromal dysplasia

The nine biopsy reports showed a heterogeneous set of alterations, yet with a common underlying theme:

- stromal hyperplasia,
- fibroepithelial lesions,
- apocrine metaplasia,
- stromal hypercellularity,
- florid or sclerosing adenosis,
- pseudoangiomatous stromal hyperplasia (PASH).

These findings are not random; they represent different expressions of a stromal dysplastic phenotype characterized by:

- abnormal proliferation,
- ECM remodeling,
- proliferative reactivity.

This pattern closely parallels the known pathophysiology of lipedema in adipose tissue of the limbs.

7. Identification of two invasive ductal carcinomas

The diagnosis of invasive carcinoma in two cases (micropapillary and ductal) cannot support statistical conclusions, but it raises important questions regarding potential associations between:

- chronic stromal proliferation,
- inflammatory microenvironment,
- ECM alterations,
- oncologic risk.

This observation highlights an area that warrants further investigation.

## 5. Conclusions

It is not surprising that a connective tissue disorder such as lipedema would also involve the breast, an organ exceptionally rich in connective tissue distributed across distinct and functionally specialized compartments. Beyond adipose tissue, which accounts for much of breast volume, the mammary gland contains specialized intralobular connective tissue and, most prominently, an extensive interlobular stroma that provides essential structural support.

The periductal (or mantellar) stroma, which closely surrounds the ductules, consists of densely organized collagen fibers with a substantial elastic component, effectively functioning as the lamina propria of the ductal system. The intralobular stroma is more delicate, containing fine reticular collagen fibrils and a ground substance rich in acidic mucopolysaccharides, which contribute to variable degrees of tissue hydration. This hydration increases in the premenstrual period, producing the well-known cyclical breast tension and discomfort (mastodynia).

Even more extensive is the interlobular connective tissue, which has no direct contact with ductal epithelium. It forms a network extending from the dermis into deeper layers, merging with the subcutaneous connective tissue. This stromal compartment can vary markedly in volume and density in response to endocrine stimuli and overall physiological state, making the breast a dynamic organ highly sensitive to connective tissue remodeling.

Within this framework, the alterations observed in our cohort—including the predominance of fibro-glandular parenchyma, diffuse cysts, microcalcifications, fibroadenomas, stromal hyperplasia, and reactive lymphadenopathy—are entirely consistent with a systemic condition affecting the extracellular matrix, fibrous components, and stromal regulation. Because the breast is one of the organs richest in modifiable connective tissue, it is a natural site for expressing a proliferative–fibrotic disorder such as lipedema. From this perspective, the presence of a characteristic breast phenotype may represent an overlooked yet physiopathologically coherent component of lipedema.

Based on the collected data, it is reasonable to hypothesize that the constellation of findings—predominantly fibro-glandular parenchyma, multiple diffuse cysts, microcalcifications, fibroadenomas, stromal hyperplasia, and lymph node reactivity—constitutes a distinctive breast pattern in women with lipedema. Although studies with control groups and larger samples are needed, this pattern may hold clinical relevance in the future. The presence of these findings, particularly when recurrent or accompanied by mastodynia, tissue instability, or systemic symptoms consistent with a connective tissue disorder, could serve as a potential indicator prompting targeted evaluation for lipedema.

Such an approach would be innovative and could support both earlier diagnosis and recognition of underexplored phenotypic dimensions of the disease. However, this hypothesis requires confirmation through prospective, controlled studies before it can be integrated into clinical practice.

## 6. Limitations

This study presents several limitations that should be considered when interpreting the results.

- Absence of a control group

We did not include a comparison sample of women without lipedema. This limits the ability to determine whether the breast alterations observed are specific to lipedema or fall within the range of variability present in the general population.

- Descriptive observational design

The study was not designed to evaluate causal relationships but rather to describe the frequency of specific structural and symptomatic patterns. Therefore, direct causal inferences between lipedema and breast alterations cannot be made.

- Non-random sample selection

Participants were women who voluntarily responded to an open invitation and who had already undergone breast imaging. This may introduce selection bias, as individuals with symptoms, concerns, pain, or prior abnormal findings are more likely to participate.

- Heterogeneity of diagnostic examinations

Breast imaging studies (mammography, ultrasound, MRI) were not performed in a single center nor using standardized protocols. Differences in operators, equipment, and reporting practices may have contributed to variability in the findings.

- Lack of centralized image review

A blinded, centralized review of imaging studies by dedicated breast radiologists was not feasible. Data were derived from available clinical reports, which may affect the homogeneity and comparability of the results.

- Possible overestimation of certain alterations

The marked fibroglandular density observed in many patients may make breast evaluation more challenging, increasing the likelihood of detecting microcalcifications, cysts, or stromal thickening. This may contribute to a relative overestimation of these findings.

- Limited number of biopsies

Only nine patients underwent biopsy, limiting the strength of conclusions regarding histological findings. The sample is insufficient to estimate prevalence or risk.

- Lack of endocrine and metabolic data

BMI values were available for only a subset of patients (31/62). Moreover, BMI is an imperfect metric in lipedema, as it does not adequately capture regional fat deposition, connective tissue alterations, or edema. This limits the interpretability and generalizability of BMI-related observations and underscores the need for more specific measures of body composition and fat distribution in future studies.

Systematic information on hormonal profile, use of hormonal therapies, or reproductive history was not collected, despite these factors potentially influencing breast structure and stromal response.

- Possible underestimation of breast pain

Pain assessment was based on self-report through questionnaire and did not employ validated scales or longitudinal measurement. This may limit the accuracy of mastodynia estimation.

- Limited generalizability

Because the sample consisted exclusively of women with a clinical diagnosis of lipedema, the results cannot be automatically generalized to all individuals with lipedema or to the general population.

## Data Availability

The data presented in this study are available on request from the corresponding author.
